# ε-Viniferin Rejuvenates Senescence via *RGS16* Regulation: In Vitro Evidence

**DOI:** 10.3390/ph18091254

**Published:** 2025-08-24

**Authors:** Ji Ho Park, Yun Haeng Lee, Kyeong Seon Lee, Yoo Jin Lee, Jee Hee Yoon, Byeonghyeon So, Duyeol Kim, Minseon Kim, Hyung Wook Kwon, Youngjoo Byun, Ki Yong Lee, Joon Tae Park

**Affiliations:** 1 Division of Life Sciences, College of Life Sciences and Bioengineering, Incheon National University, Incheon 22012, Republic of Korea; 202428002@inu.ac.kr (J.H.P.); yh.lee@inu.ac.kr (Y.H.L.); juli9709@inu.ac.kr (Y.J.L.); yoojn0905@inu.ac.kr (J.H.Y.); tundra@inu.ac.kr (B.S.); papaya1130@inu.ac.kr (D.K.); alstjs0323@inu.ac.kr (M.K.); hwkwon@inu.ac.kr (H.W.K.); 2 College of Pharmacy, Korea University, Sejong 30019, Republic of Korea; kslee0118@korea.ar.kr (K.S.L.); yjbyun1@korea.ac.kr (Y.B.); 3 Interdisciplinary Major Program in Innovative Pharmaceutical Sciences, Korea University, Sejong 30019, Republic of Korea; 4 Convergence Research Center for Insect Vectors, Incheon National University, Incheon 22012, Republic of Korea

**Keywords:** reactive oxygen species (ROS), ε-viniferin, RGS16, senolytic, senescence rejuvenation

## Abstract

**Background**: Reactive oxygen species (ROS) generated due to mitochondrial dysfunction are one of the primary causes of the initiation and progression of senescence. Although reducing mitochondrial ROS production is known as an effective strategy for the treatment of aging, effective components that reduce mitochondrial ROS production or effective treatments that utilize them have not yet been developed. **Methods**: Screening of plant-generated secondary metabolites to overcome ROS-mediated stress found that ε-viniferin, a dimer of resveratrol, effectively reduces mitochondrial ROS production. **Results**: ε-viniferin induced efficient electron transport and reduced mitochondrial ROS, a consequence of inefficient electron transport. In addition, ε-viniferin acted as a senolytic that selectively eliminates senescent fibroblasts, thereby restoring mitochondrial function and senescence-associated phenotypes. RNA sequencing analysis revealed that *regulator of G protein signaling 16* (*RGS16*) was an important gene for ε-viniferin-mediated senescence rejuvenation. Upregulation of *RGS16* showed similar effects as ε-viniferin in reducing mitochondrial ROS production and restoring mitochondrial function. **Conclusions**: This study discovered a novel mechanism by which ε-viniferin rejuvenates senescence by lowering ROS production in mitochondria. The novel mechanism will serve as a basis for developing therapeutics that regulate mitochondrial ROS production to treat aging.

## 1. Introduction

Senescence is defined as a decrease in mitotic ability due to various stressors, including DNA damage, oxidative stress, and telomere dysfunction [1]. Cell cycle arrest, the senescence-associated secretory phenotype, and lipofuscin accumulation are all hallmarks of senescence [2]. A decline in the composition and functionality of organelles is another hallmark of senescence [2]. Mitochondria are one of the organelles that undergo the greatest structural and functional changes during senescence [3]. Mitochondrial dysfunction causes electron leak in the electron transport chain (ETC), which produces reactive oxygen species (ROS) in the complexes that make up the ETC. ROS are chemically reactive molecules, including hydrogen peroxide, superoxide anions, and hydroxyl radicals. Physiological levels of ROS are essential for homeostasis, signaling pathway, and pathogen defense [4]. However, ROS exceeding physiological levels are detrimental to cellular organelles [4]. In addition to being the primary organelles that produce ROS, mitochondria are also the organelles that are directly damaged by ROS. Oxidative damage to mitochondria by ROS has been known to be one of the main causes of senescence [5]. Therefore, therapeutic options that regulate mitochondrial ROS levels have emerged as a key strategy in anti-aging research. However, there is currently no known effective method to prevent senescence by reducing mitochondrial ROS levels, and further research in this area is needed.

Oxidative stress caused by ROS is a main cause of aging. For example, polyADP-ribose polymerase 1, a nuclear enzyme that degrades nicotinamide adenine dinucleotide (NAD^+^), is activated by excessive ROS [6,7]. Increased NAD^+^ utilization by PADP-ribose polymerase 1 leads to a rapid decrease in NAD^+^ levels, thereby accelerating aging [8,9,10]. The discovery of *mev-1* (a complex II ortholog) mutations in the *Caenorhabditis elegans* provides further proof that oxidative stress plays a crucial role in aging. *mev-1* mutants generate twice as much ROS in mitochondria as the wild-type [11]. Elevated levels of ROS within mitochondria in *mev-1* mutants induce age-dependent physiological changes [12,13]. Results from animals deficient in *superoxide dismutase 1* (*SOD1*), a protein present in the mitochondrial intermembrane space and matrix, provide further proof that oxidative stress plays a key role in aging [14]. *SOD1* deficiency leads to oxidative damage and increased superoxide anion production, which contributes to premature loss of skeletal muscle mass [14]. Considering these causal relationships, reducing oxidative stress caused by ROS may be one of the promising aging treatments.

Plants produce secondary metabolites to respond to pathogens and various stresses. Terpenes, phenols, and nitrogen-containing chemicals are the three major secondary metabolites produced by plants [15]. Their chelating and free radical scavenging properties play a crucial role in the response of plants to pathogens and various stresses, and these roles are exploited to create pharmaceuticals and food additives [15]. For example, phillyrin is known as a polyphenol chemical with anti-inflammatory and anti-tumor activities [16] (Table 1). Rosamultin, a terpene component isolated from *Potentilla anserina*, exhibits potent antioxidant properties [17] (Table 1). ε-viniferin, a type of stilbenoid and polyphenol, is known to have antioxidant properties [18] (Table 1). Although the effects of these secondary metabolites are encouraging, further studies are necessary to decide whether they can actually reduce mitochondrial ROS in senescent fibroblasts.

*RGS16* acts as a GTPase activating protein, which catalyzes the hydrolysis of GTP attached to the G protein alpha subunit [19]. Recent studies have identified that abnormally increased expression of *RGS16* in esophageal squamous cell carcinoma *RGS16* promotes cancer progression by affecting cell proliferation [20]. Moreover, *RGS16* was upregulated in gastric cancer, and overexpression of *RGS16* showed the effect of reducing elevated ROS [21]. However, no study has examined the role of *RGS16* on reduction in mitochondrial ROS levels and recovery in mitochondrial function.

The most widely used reagent for detecting mitochondrial ROS is dihydrorhodamine 123 (DHR123). Rhodamine 123, a dye that specifically stains mitochondria, is reduced to form DHR123 [22]. DHR123 binds to mitochondrial hydroxyl radicals and is oxidized to cationic rhodamine 123, providing a measure of mitochondrial ROS levels [23].

In this study, we found that ε-viniferin was the major secondary metabolite that reduced mitochondrial ROS production. A reduction in mitochondrial ROS production by ε-viniferin restored mitochondrial function, thereby rejuvenating senescence-associated phenotypes. Through transcriptome analysis, we elucidated the underlying mechanism by which ε-viniferin restores mitochondrial function through the regulation of *RGS16*. Here, we propose a new mechanism by which ε-viniferin restores senescence, which is expected to be utilized as a new treatment for aging or aging-related diseases.

## 2. Results

### 2.1. ε-Viniferin Reduces Mitochondrial ROS Levels

**Figure 1:**
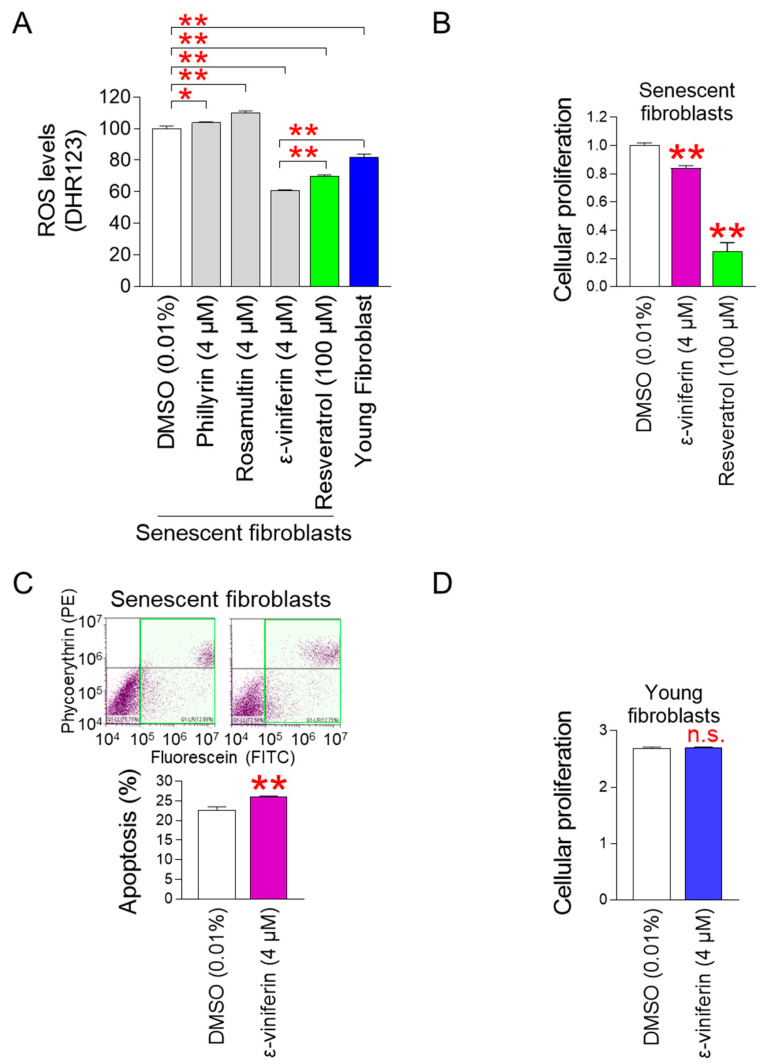
ε-viniferin reduces mitochondrial ROS levels. (**A**) Mitochondrial ROS levels were assessed in senescent fibroblasts administered DMSO (0.01%), phillyrin (4 μM), rosamultin (4 μM), ε-viniferin (4 μM), or resveratrol (100 μM) every 4 days for 12 days. Dihydrorhodamine 123 (DHR123) was used to measure the amount of ROS in the mitochondria. Young fibroblasts were employed as a positive control. * *p* < 0.05, ** *p* < 0.01, Mann–Whitney U test. Mean ± S.D., *n* = 3. (**B**) Cell proliferation was assessed in senescent fibroblasts administered DMSO (0.01%), ε-viniferin (4 μM), or resveratrol (100 μM) every 4 days for 12 days. Water-soluble tetrazolium salt (WST) assay was used to measure cell proliferation. ** *p* < 0.01, Mann–Whitney U test. Mean ± S.D., *n* = 3. (**C**) Apoptosis was assessed in senescent fibroblasts administered DMSO (0.01%) or ε-viniferin (4 μM) at 4-day intervals for 12 days. The FITC Annexin V Apoptosis Detection Kit was utilized to measure the apoptosis rate. Apoptotic cell populations are indicated by green squares. ** *p* < 0.01, Mann–Whitney U test. Mean ± S.D., *n* = 3. (**D**) Cell proliferation was assessed in young fibroblasts administered DMSO (0.01%) or ε-viniferin (4 μM) every 4 days for 12 days. WST assay was used to measure cell proliferation. not significant (n.s.), Mann–Whitney U test. Mean ± S.D., *n* = 3.

Secondary metabolites (phillyrin, rosamultin, ε-viniferin) were utilized to identify metabolites that could effectively reduce ROS production in mitochondria (Table 1). The three metabolites were administered to senescent fibroblasts at 4 µM for 12 days to evaluate the effect of each substance on mitochondrial ROS production. Then, the amount of mitochondrial hydroxyl radicals was measured using DHR123 [22]. Resveratrol, known as the most potent antioxidant among secondary metabolites, was used as a positive control [24]. Young fibroblasts were employed as an additional positive control. Compared with the DMSO-treated senescent fibroblasts, resveratrol significantly reduced mitochondrial hydroxyl radicals in senescent fibroblasts (Figure 1A). Moreover, compared with the DMSO-treated senescent fibroblasts, young fibroblasts showed the significant reduction in mitochondrial hydroxyl radicals (Figure 1A). However, the mitochondrial hydroxyl radical in senescent fibroblasts were increased by phillyrin, rosamultin, which are known to have antioxidant properties (Figure 1A). On the contrary, ε-viniferin significantly reduced mitochondrial hydroxyl radicals in senescent fibroblasts compared with the DMSO-treated senescent fibroblasts (Figure 1A). Furthermore, compared with the positive control (resveratrol and young fibroblasts), ε-viniferin significantly reduced mitochondrial hydroxyl radicals (Figure 1A). These results demonstrate that among three metabolites, ε-viniferin is effective in reducing mitochondrial ROS levels, and its efficacy is superior to that of resveratrol, a potent antioxidant.

ε-viniferin is a resveratrol dimer composed of two resveratrol molecules. Since resveratrol has a senolytic effect that selectively removes senescent cells [25], we examined whether ε-viniferin has the same effect. Resveratrol significantly decreased the proliferation of senescent fibroblasts compared with the DMSO control (Figure 1B). Moreover, ε-viniferin significantly decreased the proliferation of senescent fibroblasts compared with the DMSO control (Figure 1B). These findings imply that ε-viniferin functions as a senolytic by preventing senescent fibroblasts from proliferating.

The discovery that ε-viniferin inhibited the proliferation of senescent fibroblasts led us to determine whether ε-viniferin, like resveratrol, eliminates senescent fibroblasts through apoptosis. Compared with the DMSO control, the 4 μM ε-viniferin significantly increased the apoptosis rate (Figure 1C; green square). These results demonstrate that ε-viniferin acts as a senolytic by inducing apoptosis in senescent fibroblasts.

The finding that ε-viniferin acts as a senolytics led us to re-examine whether ε-viniferin has senolytic properties that affect only senescent fibroblasts but not non-senescent fibroblasts. The impact of ε-viniferin on the proliferation of young fibroblasts was observed. The proliferation of young fibroblasts was unaffected by 4 μM ε-viniferin when compared with the DMSO control (Figure 1D). These results demonstrate that ε-viniferin acts as a senolytic without affecting the proliferation of young fibroblasts.

### 2.2. The Mitochondrial Function Is Restored by ε-Viniferin

#### 2.2.1. The Increase in OXPHOS Efficiency by ε-Viniferin

**Figure 2:**
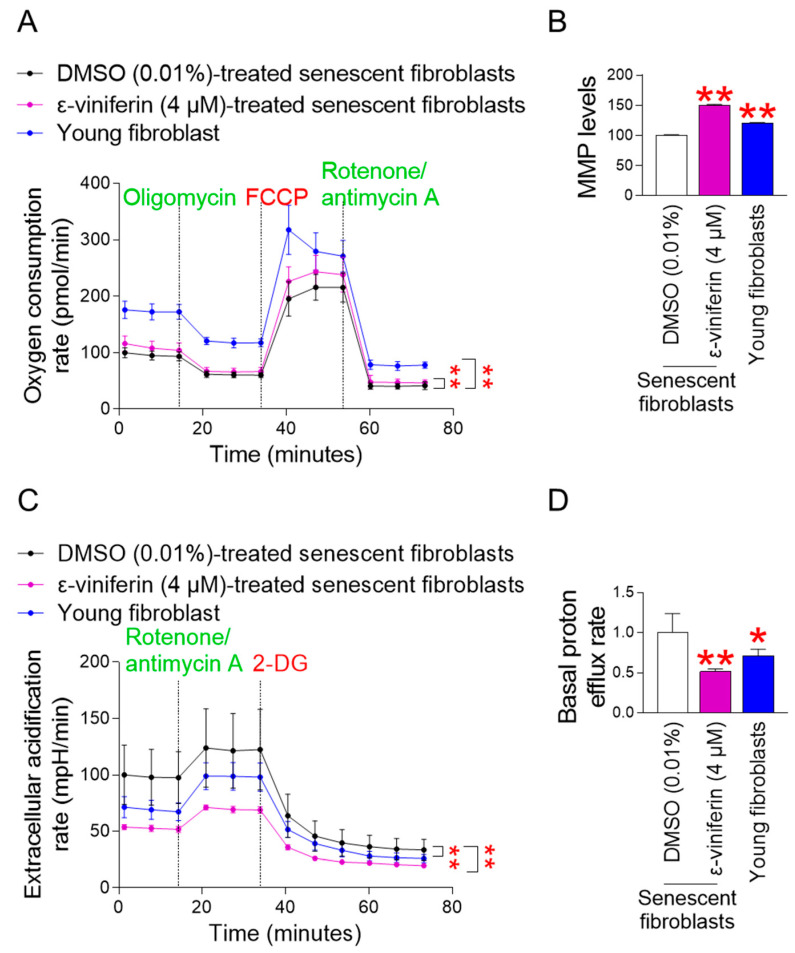
The mitochondrial function is restored by ε-viniferin. (**A**) Oxygen consumption rate (OCR; pmol/min) was assessed in senescent fibroblasts treated with DMSO (0.01%) or ε-viniferin (4 μM) every 4 days for 12 days. Young fibroblasts were utilized as a positive control. The OCR was measured using the Seahorse XF Cell Mito Stress Test Kit. ** *p* < 0.01, two–way ANOVA followed by Bonferroni’s post hoc test. Means ± S.D., *n* = 5. (**B**) MMP levels were assessed in senescent fibroblasts administered DMSO (0.01%) or ε-viniferin (4 μM) every 4 days for 12 days. Young fibroblasts were utilized as a positive control. JC-10 was used to measure MMP levels. ** *p* < 0.01, Mann–Whitney U test. Mean ± S.D., *n* = 3. (**C**) Extracellular acidification rate (ECAR; mpH/min) was assessed in senescent fibroblasts treated with DMSO (0.01%) or ε-viniferin (4 μM) every 4 days for 12 days. Young fibroblasts were utilized as a positive control. To quantify the ECAR, the Seahorse XF Glycolytic Rate Assay Kit was utilized. ** *p* < 0.01, two–way ANOVA followed by Bonferroni’s post hoc test. Means ± S.D., *n* = 5. (**D**) Basal proton efflux rate was assessed in senescent fibroblasts administered DMSO (0.01%) or ε-viniferin (4 μM) every 4 days for 12 days. Young fibroblasts were utilized as a positive control. The basal proton efflux rate was measured using the Seahorse XF Glycolytic Rate Assay Kit. * *p* < 0.05, ** *p* < 0.01, Mann–Whitney U test. Mean ± S.D., *n* = 5.

Inefficiency in electron transport in the ETC is one of the primary causes of ROS generation in mitochondria [26]. Specifically, inefficient electron transport within the ETC reduces oxygen to superoxide radicals in the ETC complexes [26]. Oxidative phosphorylation (OXPHOS) efficiency is one of the means to measure the efficiency of electron transport [27]. To reveal the mechanism of ROS reduction by ε-viniferin [28], the OXPHOS efficiency was examined by measuring the oxygen consumption rate (OCR) [28]. OCR was measured sequentially after oligomycin, carbonyl cyanide-p-trifluoromethoxyphenylhydrazone (FCCP), and rotenone/antimycin A administration [29]. Specifically, non-mitochondrial respiration can be assessed after oligomycin administration, maximal respiration after FCCP, and ATP-coupled respiration after rotenone/antimycin A. Young fibroblasts were employed as a positive control. Young fibroblasts exhibited significantly higher OCR values than DMSO-treated senescent fibroblasts (Figure 2A; black vs. blue lines). Moreover, ε-viniferin significantly upregulated OCR values compared to DMSO-treated senescent fibroblasts, indicating an increase in OXPHOS efficiency (Figure 2A; black vs. purple lines). The increase in OXPHOS efficiency by ε-viniferin indicates efficient electron transport, suggesting that this might be the mechanism of mitochondrial ROS reduction by ε-viniferin.

#### 2.2.2. The Increase in MMP Levels by ε-Viniferin

Efficient electron transport in the ETC generates an electrochemical gradient of proton ions by ETC complexes [5]. The electrical potential differential between the mitochondrial matrix and the intermembrane space is known as the mitochondrial membrane potential (MMP) [30]. The discovery of efficient electron transport by ε-viniferin led us to examine changes in MMP, a marker of mitochondrial functional recovery. Young fibroblasts exhibited significantly increased MMP levels compared to DMSO-administered senescent fibroblasts (Figure 2B). In senescent cells treated with ε-viniferin, MMP levels were significantly higher than those in the DMSO-administered senescent fibroblasts, suggesting the restoration of mitochondrial function by ε-viniferin (Figure 2B).

#### 2.2.3. The Decrease in the Glycolysis Rate by ε-Viniferin

MMP is a motive force that makes it possible for OXPHOS to produce ATP [31]. Increased intracellular ATP production by OXPHOS reduces the reliance on glycolysis as an intracellular energy source. The discovery of ε-viniferin-mediated increase in OXPHOS and MMP led us to examine the glycolysis rate by measuring the extracellular acidification rate (ECAR) [28]. ECAR was measured before/after rotenone/antimycin A administration and after 2-deoxy-D-glucose (2-DG) [28]. Specifically, the basal glycolysis rate before rotenone/antimycin A administration, glycolysis rate after rotenone/antimycin A, and post-2-DG acidification after 2-DG were measured. Young fibroblasts had significantly lower ECAR values than DMSO-treated senescent fibroblasts (Figure 2C; black vs. blue lines). Moreover, ε-viniferin significantly downregulated ECAR values compared with DMSO-treated senescent fibroblasts, indicating that ε-viniferin downregulated the glycolysis rate (Figure 2C; black vs. purple lines).

#### 2.2.4. The Decrease in the Basal Proton Efflux Rate by ε-Viniferin

Lactate is produced through an inefficient energy metabolism pathway after glycolysis [32]. Protons are produced during the conversion of pyruvate to lactate [33]. To determine whether ε-viniferin affects the post-glycolytic pathway, the basal proton efflux rate was examined. Young fibroblasts had a significantly lower basal proton efflux rate than DMSO-administered senescent fibroblasts (Figure 2D). Moreover, senescent fibroblasts treated with ε-viniferin had a significantly lower basal proton efflux rate than DMSO-administered senescent fibroblasts (Figure 2D). These results suggest that ε-viniferin blocks the inefficient energy metabolism pathway by restoring mitochondrial function.

### 2.3. ε-Viniferin Activates Mitophagy to Remove Damaged Mitochondria

**Figure 3:**
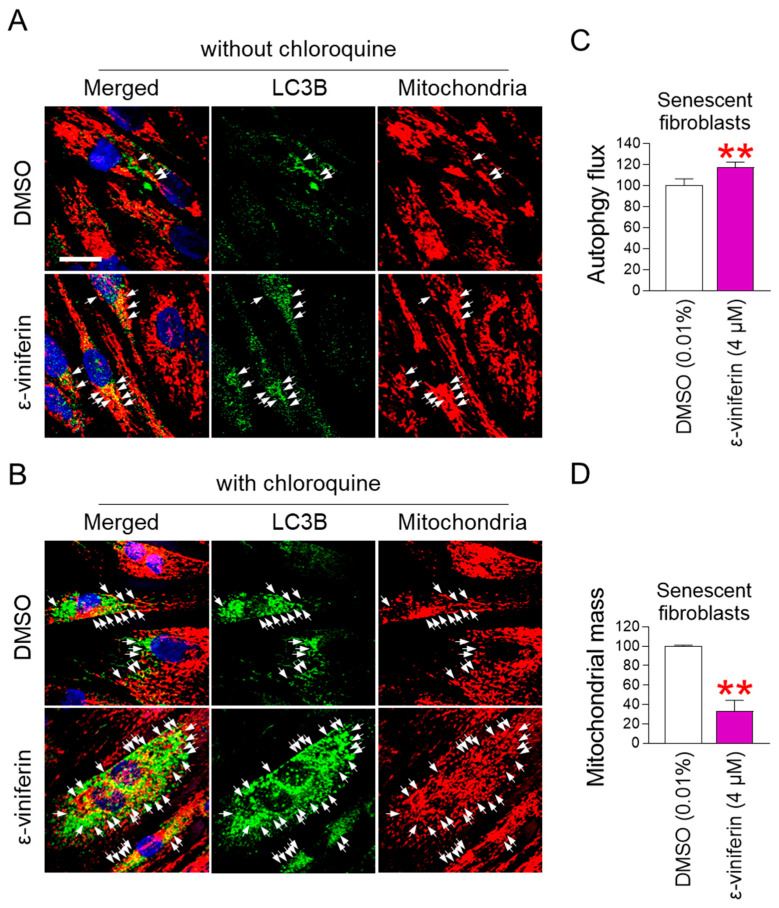
ε-viniferin activates mitophagy to remove damaged mitochondria. (**A**,**B**) Immunostaining for LC3B (green) and mitochondria (red). Nuclei stained with 4′,6-diamidino-2-phenylindole (DAPI) are shown in blue. Senescent fibroblasts were treated with DMSO (0.01%) or ε-viniferin (4 μM) every 4 days for 12 days. Cells were added without or with 20 μM chloroquine 24 h before immunofluorescence staining. Scale bar 10 μm. Mitophagy is indicated by a white arrow. Figure S1 displays full-size immunofluorescence pictures. (**C**,**D**) Autophagy flux or mitochondrial mass was assessed in senescent fibroblasts administered DMSO (0.01%) or ε-viniferin (4 μM) every 4 days for 12 days. Cyto-ID staining solution and MitoTracker^TM^ Deep Red FM Dye (MTDR) were used to measure autophagy flux and mitochondrial mass, respectively. ** *p* < 0.01, Mann–Whitney U test. Mean ± S.D., *n* = 3.

The discovery of ε-viniferin-mediated restoration of mitochondrial function raised questions about the mechanism by which ε-viniferin achieves these effects. Mitophagy removes damaged mitochondria and restores mitochondrial function [34]. Therefore, we hypothesized that ε-viniferin restores mitochondrial function by activating mitophagy. Since mitophagy specifically removes defective mitochondria using autophagosomes [34], we investigated the colocalization of mitochondria with microtubule-associated protein 1A/1B-light chain 3B (LC3B), a membrane protein of autophagosomes [35]. Colocalization between LC3B and mitochondria was rarely noticeable in DMSO-administered senescent fibroblasts (Figure 3A; white arrows). However, colocalization of LC3B and mitochondria reappeared after ε-viniferin treatment (Figure 3A; white arrows). To further confirm the role of ε-viniferin in mitophagy, a group treated with chloroquine (CQ) was included. CQ induces autophagosome accumulation by disrupting lysosomal pH, which increases colocalization of LC3B and mitochondria [36]. The CQ-treated group showed increased colocalization compared to the non-CQ-treated group, providing proof-of-concept for the effectiveness of CQ (Figure 3A,B, white arrows). Specifically, the group co-treated with CQ and ε-viniferin showed increased colocalization compared to the group co-treated with CQ and DMSO (Figure 3B; white arrows). These results suggest that ε-viniferin promotes mitophagy activation.

Next, to quantify mitophagy activation by ε-viniferin, autophagic flux was measured. Autophagy flux is the rate at which autophagy removes damaged organelles [37]. Senescent fibroblasts treated with ε-viniferin exhibited a significant increase in autophagic flux compared with the DMSO control (Figure 3C). Since autophagic flux is the rate of the removal of organelles, including damaged mitochondria, mitochondrial mass was measured to determine whether mitophagy removes damaged mitochondria. Senescent fibroblasts treated with ε-viniferin had a significant reduction in mitochondrial mass compared with the DMSO control, suggesting the activation of mitophagy (Figure 3D).

### 2.4. ε-Viniferin Rejuvenates Senescence-Associated Phenotypes

**Figure 4:**
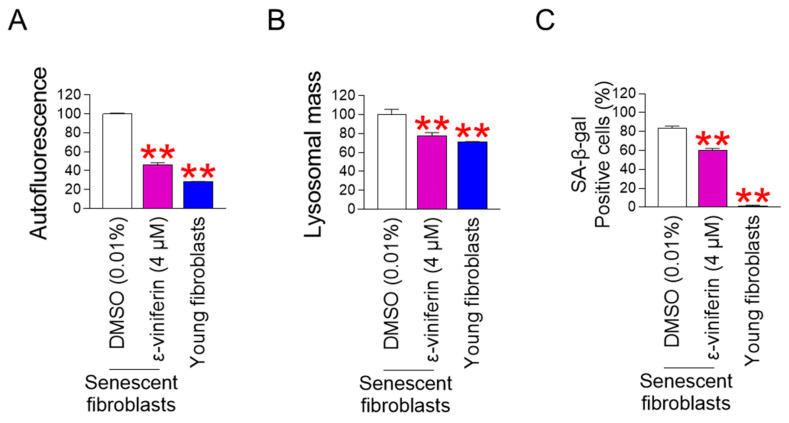
ε-viniferin rejuvenates senescence-associated phenotypes. Autofluorescence (**A**), lysosomal mass (**B**), or SA-β-gal staining (**C**) were assessed in senescent fibroblasts treated with DMSO (0.01%) or ε-viniferin (4 μM) every 4 days for 12 days. Young fibroblasts were utilized as a positive control. Medium without dye, LysoTracker^TM^ Deep Red (LTDR), and SA-β-gal staining solution were used to measure autofluorescence, lysosomal mass, and SA-β-gal-positive cells, respectively. ** *p* < 0.01, Mann–Whitney U test. Mean ± S.D., *n* = 3.

Mitochondrial functional recovery is the prerequisite for regenerating senescent cells into young cells [38,39,40,41,42]. The discovery that ε-viniferin restores mitochondrial function prompted us to evaluate how ε-viniferin affects senescence. Lipofuscin, a cross-linked protein residue generated by iron-catalyzed oxidation during the senescence process, accumulates in lysosomes [43]. Therefore, we investigated how ε-viniferin affects the level of lipofuscin. The intracellular accumulation of lipofuscin was assessed by measuring autofluorescence [44]. Young fibroblasts showed significantly decreased autofluorescence compared with DMSO-treated senescent fibroblasts (Figure 4A). Moreover, autofluorescence in ε-viniferin-treated senescent fibroblasts was significantly reduced compared with the DMSO-treated senescent fibroblasts, suggesting that ε-viniferin efficiently inhibits lipofuscin accumulation (Figure 4A).

Lysosomes filled with lipofuscin serve as a storage site for newly produced hydrolytic enzymes, which results in decreased activity of hydrolytic enzymes and consequently decreased lysosomal activity [45]. To compensate for the decreased activity, lysosomal mass increases [45]. Lysosomal mass was examined to ascertain whether ε-viniferin influences lysosomal activity. Young fibroblasts showed significantly decreased lysosomal mass compared to DMSO-treated senescent fibroblasts (Figure 4B). Moreover, fibroblasts treated with ε-viniferin showed a significant decrease in lysosomal mass compared with DMSO-treated senescent fibroblasts, suggesting that the decrease in lipofuscin accumulation led to an increase in lysosomal activity (Figure 4B).

Next, we examined how ε-viniferin affects senescence-associated beta-galactosidase (SA-β-gal), a hallmark of senescence [46]. Young fibroblasts showed a significantly lower percentage of SA-β-gal-positive cells than DMSO-treated senescent fibroblasts (Figure 4C). Moreover, senescent fibroblasts treated with ε-viniferin exhibited a significantly lower percentage of SA-β-gal-positive cells than DMSO-treated senescent fibroblasts, indicating the ε-viniferin-mediated senescence amelioration (Figure 4C).

### 2.5. RGS16 Regulates ε-Viniferin-Mediated Mitochondrial ROS Reduction

**Figure 5:**
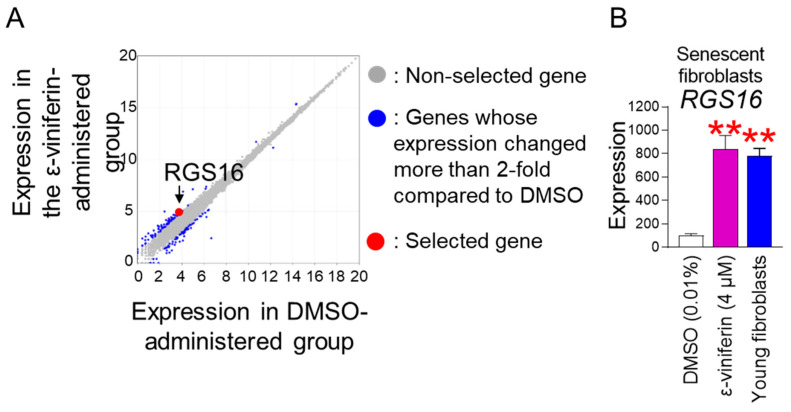
*RGS16* regulates ε-viniferin-mediated mitochondrial ROS reduction. (**A**) Transcriptome expression profiling was assessed in senescent fibroblasts treated with DMSO (0.01%) or ε-viniferin (4 μM) every 4 days for 12 days. DEG analysis identified 31 genes whose expression levels were altered more than 2-fold compared to the DMSO control (blue dots). *RGS16* was selected as a key candidate as it is known to suppress the worsening of oxidative stress (red dot). Non-selected genes (gray dots). (**B**) *RGS16* expression levels were assessed in senescent fibroblasts treated with DMSO (0.01%) or ε-viniferin (4 μM) at 4-day intervals for 12 days. Young fibroblasts were employed as a positive control. Quantitative PCR (qPCR) was used to measure *RGS16* expression levels. ** *p* < 0.01, Mann–Whitney U test. Mean ± S.D., *n* = 3.

The discovery that ε-viniferin has senescence-rejuvenating properties led us to investigate the genes regulated by ε-viniferin to induce these effects. We analyzed the underlying signaling pathway by RNA sequencing using senescent fibroblasts treated with DMSO or ε-viniferin. Transcriptome expression profiling led to the discovery of differentially expressed genes (DEGs). DEG analysis identified 31 genes with levels that were more than 2-fold altered compared with the DMSO control (Figure 5A and Table S1; blue dots). Among these 31 genes, a candidate gene approach was performed to select candidate genes involved in ROS production or inhibition. We selected the *regulator of G protein signaling 16* (*RGS16*; accession number: NM_002928), which counteracts the exacerbation of oxidative stress [21] (Figure 5A and Table S1; red dot). DEG analysis showed that senescent fibroblasts treated with ε-viniferin showed a 2.56-fold increase in RGS16 expression compared with the DMSO control (Figure 5A and Table S1; red dot). Then, the result was further validated by quantitative PCR (qPCR). Young fibroblasts, used as a positive control, showed significantly higher *RGS16* expression than DMSO-treated senescent fibroblasts (Figure 5B). Moreover, the expression of *RGS16* was significantly higher in senescent fibroblasts treated with ε-viniferin than in DMSO-treated senescent fibroblasts, indicating that *RGS16* is a candidate gene regulated by ε-viniferin (Figure 5B).

### 2.6. RGS16 Upregulation Restores Mitochondrial Function

**Figure 6:**
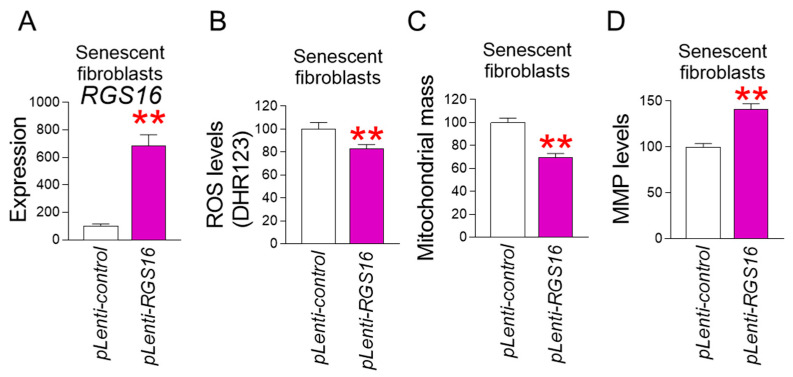
*RGS16* upregulation restores mitochondrial function. The expression levels of *RGS16* (**A**), mitochondrial ROS levels (**B**), mitochondrial mass (**C**), or MMP levels (**D**) were assessed in senescent fibroblasts transduced with lentivirus expressing pLenti-control or pLenti-*RGS16*. Quantitative PCR (qPCR), Dihydrorhodamine 123 (DHR123), MitoTracker^TM^ Deep Red FM Dye (MTDR), and JC-10 were used to measure *RGS16* expression levels, mitochondrial ROS levels, mitochondrial mass and MMP levels, respectively. ** *p* < 0.01, Mann–Whitney U test. Mean ± S.D., *n* = 3.

The discovery that *RGS16* is a candidate gene regulated by ε-viniferin prompted us to study whether upregulation of *RGS16* could restore mitochondrial function similar to ε-viniferin. Senescent fibroblasts were infected with lentivirus expressing *RGS16* or a control lentivirus. Senescent fibroblasts infected with lentivirus producing *RGS16* had significantly higher *RGS16* expression than the control group (Figure 6A).

Next, we examined the effect of RGS16 overexpression on mitochondrial ROS levels. Senescent fibroblasts infected with lentivirus producing *RGS16* had significantly lower mitochondrial ROS levels than the control group (Figure 6B). These results imply that *RGS16* upregulation exhibits the same ROS-reducing effect as ε-viniferin.

We then looked at the impact of *RGS16* overexpression on mitochondrial mass since ROS-induced mitochondrial damage causes an increase in mitochondrial mass [47,48]. Senescent fibroblasts infected with lentivirus producing *RGS16* had significantly lower mitochondrial mass than the control group (Figure 6C). These results imply that *RGS16* upregulation exhibits the same mitochondrial mass-reduction effect as ε-viniferin.

The discovery that ε-viniferin reduced mitochondrial ROS and mitochondrial mass led us to investigate whether upregulation of *RGS16* could induce an increase in MMP, an indicator of mitochondrial function recovery. Senescent fibroblasts infected with lentivirus producing *RGS16* had significantly higher MMP than the control group (Figure 6D). These results suggest that upregulation of *RGS16* has the same mitochondrial functional recovery effect as ε-viniferin.

## 3. Discussion

The progression of senescence is significantly influenced by damage to cellular organelles, particularly mitochondria, due to ROS-mediated oxidative stress [49]. Mitochondria, responsible for most cellular oxygen consumption and ATP production, generate ROS as a byproduct [50]. Specifically, superoxide, generated by complexes I and III of the ETC, is a major source of ROS [50]. Senescence-related decline in mitochondrial function, including reduced ETC activity, leads to increased superoxide production, further damaging the ETC and accelerating senescence [51,52]. This creates a cycle of worsening mitochondrial damage and increased ROS production, ultimately accelerating the senescence process. Reducing mitochondrial ROS production is therefore considered a key strategy for potentially reversing or slowing down senescence. Here, we found that ε-viniferin reduces mitochondrial ROS levels. Moreover, we found that the mechanism of reducing mitochondrial ROS levels is the induction of efficient electron transport in the ETC by ε-viniferin, which is evidenced by an increase in OXPHOS efficiency. This contributes to reducing ROS generation in mitochondria by reducing electron leak in the mitochondrial ETC [53]. Furthermore, a reduction in mitochondrial ROS by ε-viniferin restored mitochondrial function, which was supported by the increase in mitochondrial MMP and the decrease in dependence on glycolysis. In conclusion, this study discovered the mechanism by which ε-viniferin reduces ROS production in mitochondria, which will be a cornerstone for future strategies to treat aging through a reduction in mitochondrial ROS.

Senolytics and senomorphics are two types of senotherapies used to treat aging [54]. Senolytics are therapies that specifically kill senescent cells that negatively affect the function of non-senescent cells [54]. Senomorphics are therapies that modulate the characteristics of senescent cells to that of young cells [54]. Senomorphics have shown promise in treating aging because they change senescent cells into young cells with high proliferative capacity. However, the process by which senescent cells are changed into young cells by senomorphics is similar to the pathways that cause cancer, which may lead to unintended consequences [55]. In contrast, senolytics are therapies that specifically remove senescent cells, which may reduce the side effects that senomorphics may cause [56]. One of the most widely used senolytics is quercetin, which inhibits *B-cell lymphoma 2* (*BCL2*) [57]. *BCL2* functions to prevent programmed cell death of senescent cells, allowing them to accumulate in the body. However, quercetin induces programmed cell death of senescent cells by inhibiting *BCL2* signaling [57]. Subsequently, quercetin eliminated senescent cells, dramatically reducing inflammatory cytokines and SA-β-gal-positive cells [57]. Another *BCL2* pathway inhibitor, navitoclax, inhibits the *BCL2* pathway and activates *p53* to induce programmed cell death of senescent cells [58]. Oral administration of navitoclax induced specific elimination of senescent cells in aged mice, thereby treating age-related diseases and disorders [58]. Despite the positive effects of senolytics, there are concerns that eliminating senescent cells could reduce the body’s ability to regenerate, which would accelerate the buildup of senescent cells [59]. Therefore, there is an increasing need for novel senolytics that kill only senescent cells while maintaining normal regenerative capacity. Here, we found that ε-viniferin selectively eliminates senescent fibroblasts through apoptosis. The significance of ε-viniferin as a senolytic was highlighted by the finding that selective elimination of senescent fibroblasts by ε-viniferin did not inhibit the proliferation of young fibroblasts. To our knowledge, this is the first study to reveal the potential of ε-viniferin as a senolytic. Further studies are needed to confirm whether ε-viniferin selectively destroys senescent fibroblasts without impairing their regenerative capacity through in vivo studies using mice.

Plants produce secondary metabolites containing compounds with antioxidant activity, such as phenolic compounds, terpenoids, and alkaloids, to protect against environmental stress [60]. Representative examples of these compounds are curcumin, quercetin, and resveratrol [61]. Curcumin is a polyphenol with many phenolic hydroxyl groups, which can effectively suppress oxidative stress and neutralize free radicals [62,63]. Quercetin is a polyphenol with multiple hydroxyl groups that can donate hydrogen atoms to ROS and neutralize them and is known as an effective antioxidant [64]. Resveratrol is a polyphenol-based antioxidant produced by plants to protect themselves from pests and fungi and contains hydroxyl groups that help in scavenging radicals [62,63]. In this study, we found that among the three secondary metabolites, only ε-viniferin could effectively reduce mitochondrial ROS levels. These results may be due to the structural characteristics of ε-viniferin compared to other secondary metabolites. Since hydroxyl groups with two lone pairs of electrons on oxygen help stabilize ROS [65], we investigated the structures of phillyrin, rosamultin, and ε-viniferin considering the number of hydroxyl groups and the functional groups to which the hydroxyl groups are attached. Phillyrin has four hydroxyl groups attached to the glucose moiety (Table 1). Rosamultin has two hydroxyl groups attached to the triterpenoid nucleus and four hydroxyl groups attached to the glucose moiety (Table 1). ε-viniferin has five hydroxyl groups attached to the phenol ring (Table 1). The hydroxyl group on the glucose moiety is nucleophilic, allowing glucose to participate in various chemical reactions, such as glycosylation and esterification [66]. It also participates in redox reactions that stabilize ROS, but this reaction is possible only at the C1 position in the open-chain form [67]. Furthermore, the hydroxyl group on the triterpenoid nucleus serves as an attachment point for glycosylation, esterification, and other modifications rather than for redox reactions [68]. However, the hydroxyl group on the phenolic ring is an active group that increases the electron density of the aromatic ring, increasing its susceptibility to reactions with ROS [69]. Thus, ε-viniferin, with its five hydroxyl groups in the phenolic ring, exhibits a greater antioxidant capacity to reduce mitochondrial ROS than the other two secondary metabolites. Here, we propose that additional modifications of ε-viniferin may further enhance its antioxidant capacity, but recognize that further studies are needed to verify this hypothesis.

The antioxidant and anti-senescence effects of ε-viniferin may be the result of a multifactorial process. For instance, apigenin, a flavonoid present in a variety of vegetables, efficiently scavenges ROS due to the numerous hydroxyl groups in its C-ring [70]. The expression of genes related to oxidative stress, such as nuclear factor erythroid 2-related factor 2, is also regulated by apigenin [71]. Further supporting the significance of these findings is baicalin, a flavonoid present in *Scutellaria baicalensis*. Baicalin’s phenolic hydroxyl groups, which are capable of undergoing redox reactions, give it strong antioxidant properties [72]. Furthermore, baicalin inhibits the expression of inflammation-related genes, such as *interleukin-6* and *tumor necrosis factor-alpha*, which are essential for antioxidant mechanisms [73,74]. Here, we found that ε-viniferin, in addition to its structural properties that enable it to scavenge ROS, upregulates *RGS16* expression. Extending these results, we found that upregulating *RGS16* expression lowered mitochondrial ROS levels, restoring mitochondrial function similar to ε-viniferin. The significance of upregulating *RGS16* expression is supported by the finding that *RGS16* expression in young fibroblasts is much higher than in senescent fibroblasts. These results suggest that modulating *RGS16* gene expression might serve as a basis for a therapeutic approach to treating aging. However, the mechanism by which ε-viniferin affects *RGS16* expression has not been elucidated. Since the *RGS16* expression is known to be regulated by epigenetic, transcriptional, and post-translational mechanisms [75], future studies should focus on identifying which of the above mechanisms ε-viniferin regulates *RGS16* expression.

Grapes, particularly *Vitis vinifera*, are a major source of the resveratrol dimer ε-viniferin [76]. ε-viniferin accumulates primarily in the woody tissues of grapevines but is also present in other parts of the vine, including grapes, leaves, and buds. ε-viniferin is metabolized in the body through glucuronidation and sulfation [77]. Through this process, ε-viniferin is converted into a conjugate of glucuronidation and sulfate, which contributes to its low bioavailability [77]. Nonetheless, ε-viniferin has shown beneficial biological activities, including potential effects in the treatment of obesity and neurodegenerative diseases [76,78]. Specifically, ε-viniferin has been shown to decrease fat accumulation in cell and animal models, thereby preventing obesity-associated metabolic diseases including type 2 diabetes, dyslipidemia, and fatty liver disease [76]. ε-viniferin has also been shown to have neuroprotective effects, including dissolving amyloid beta plaques, a hallmark of Alzheimer’s disease [78]. The diverse biological activities of ε-viniferin suggest its potential as a multi-target therapeutic. Here, we found that ε-viniferin can be used as a therapeutic agent for treating senescence. Specifically, ε-viniferin significantly reduced senescence-associated phenotypes, such as lipofuscin, lysosomal mass, and SA-β-gal-positive cells. The fundamental mechanism of these anti-senescence effects was the mitochondrial functional recovery through the reduction in mitochondrial ROS production by ε-viniferin. Here, we propose that the diverse biological activities of ε-viniferin make it a promising candidate for the treatment of diseases with complex mechanisms, including aging.

In conclusion, we found that ε-viniferin is a secondary metabolite that effectively reduces mitochondrial ROS in senescent fibroblasts. The ability of ε-viniferin to reduce mitochondrial ROS production led to the restoration of mitochondrial function and senescence-related phenotypes. Furthermore, the underlying mechanism of these effects was based on the upregulation of *RGS16* expression by ε-viniferin. Our findings that overexpression of *RGS16* reduces mitochondrial ROS and restores mitochondrial function further strengthen this mechanism. Taken together, our findings suggest a novel mechanism by which ε-viniferin reduces mitochondrial ROS levels and rejuvenates senescence. The novel mechanism by ε-viniferin will be a cornerstone for the development of new therapeutics for aging-related diseases and aging. Future studies should focus on validating the efficacy of ε-viniferin in various in vivo models, including humans, in addition to in vitro models using cells.

## 4. Materials and Methods

### 4.1. Cell Culture

#### 4.1.1. Rationale to Use Human Dermal Fibroblasts

Human dermal fibroblasts have long been used as an experimental model to study aging and age-related diseases in the scientific community [79]. This is because they can be easily obtained from noninvasive skin biopsies; have a low proliferation rate, which allows them to preserve age-related damage; and exhibit age-related changes in phenotype, epigenome, and transcriptome [80,81].

#### 4.1.2. Criteria for Distinguishing Human Skin Fibroblasts into Senescent and Young Fibroblasts

Human dermal fibroblasts (PCS-201-010; ATCC, Manassas, VA, USA) were cultured according to a previous study protocol [82]. Human dermal fibroblasts were divided into senescent and young fibroblasts based on whether they doubled in number in ≥14 days or <2 days [83,84,85,86]. Human dermal fibroblasts were also divided into senescent and young fibroblasts based on whether they contained >65% and <2% SA-β-gal-positive cells, respectively [86].

#### 4.1.3. Cell Culture Methods

To maintain the concentration of natural compounds applied to the cells for 12 days, the medium was replaced every 4 days with a medium containing each natural compound diluted to 4 µM. On day 12, cells were washed with phosphate buffered saline (PBS; 21-031-CVC, Corning, Corning, NY, USA). Then, each analysis was performed according to the procedures mentioned below.

### 4.2. Preparation of Natural Compounds

Phyllirin, rosamultin, and ε-viniferin were isolated from *Osmanthus fragrans* var. *aurantiacus*, *Rosa rugosa*, and *Vitis amurensis*, respectively. The structure was identified by ^1^H– and ^13^C–NMR analyses. ^1^H– and ^13^C–NMR are included in the Supplementary Materials (Figures S2–S7).

### 4.3. Analysis of Mitochondrial ROS Using Flow Cytometry

Senescent fibroblasts were treated with DMSO (0.01%) or 4 µM natural substances (phyllirin, rosamultin, and ε-viniferin) at 4-day intervals for 12 days. Cells were then incubated for 30 min at 37 °C in a medium containing 30 µM DHR123 (10056-1; Biotium, Fremont, CA, USA). For flow cytometry analysis, a FACSCaliber instrument (BD Biosciences, Franklin Lakes, NJ, USA) was used. Mitochondrial ROS levels were calculated as [DHR123-stained FITC mean fluorescence intensity (MFI)] − [DHR123-unstained FITC MFI].

### 4.4. Cell Proliferation Assay

Senescent or young fibroblasts were treated with DMSO (0.01%) or ε-viniferin (4 µM) every 4 days for 12 days. As directed by the manufacturer, the EZ-cytox reagent (EZ-5000; DoGe Bio, Seoul, Korea) containing water-soluble tetrazolium salt (WST) was employed. Since some apoptotic cells were lost during the medium replacement (days 4 and 8) and washing (day 12), cell proliferation was only measured for the remaining cells.

### 4.5. Apoptosis Assay

Senescent fibroblasts were treated with DMSO (0.01%) or ε-viniferin (4 µM) every 4 days for 12 days. As directed by the manufacturer, the FITC Annexin V Apoptosis Detection Kit (556547; BD Biosciences) was utilized. Since some apoptotic cells were lost during the medium replacement (days 4 and 8) and washing (day 12), apoptosis rate was only measured for the remaining cells. Therefore, the loss of apoptotic cells during media replacement (days 4 and 8) and washing (day 12) was not reflected in the apoptosis measurements.

### 4.6. Analysis of Oxygen Consumption Rate (OCR), Extracellular Acidification Rate (ECAR), and Basal Proton Efflux Rate

Senescent fibroblasts were treated with DMSO (0.01%) or ε-viniferin (4 µM) every 4 days for 12 days. The Seahorse XF Cell Mito Stress Test Kit (101706-100; Seahorse Bioscience, Billerica, MA, USA) was used to measure OCR. The Seahorse XF Glycolytic Rate Assay Kit (103344–100; Seahorse Bioscience) was used to assess ECAR and basal proton efflux rates. OCR and ECAR were measured using a Seahorse Bioscience XFe96 flux analyzer. Briefly, 5 × 10^4^ cells were seeded per well of an XFe96 cell culture plate in an XF96 FluxPak (103793-100; Seahorse Bioscience) and incubated for 16 h in an incubator containing 5% CO_2_ at 37 °C. The medium was then replaced with glucose-free XF Assay medium (102365-100; Seahorse Bioscience) and incubated for an additional 1 h in the same incubator. OCR and ECAR were then measured, with OCR expressed as pmoles/min and ECAR as mpH/min. Data were collected from five biological replicates.

### 4.7. Flow Cytometric Analysis of MMP, Mitochondrial Mass, Lysosomal Mass, Autofluorescence, and Autophagic Flux

Senescent fibroblasts were treated with DMSO (0.01%) or ε-viniferin (4 µM) at 4-day intervals for 12 days. MMP, mitochondrial mass, and lysosomal mass were measured by treating senescent fibroblasts in medium containing each dye for 30 min at 37 °C (Table 2). Autofluorescence was measured by exposing senescent fibroblasts to the medium without any dye for 30 min at 37 °C. Autophagic flux was assessed by applying (w/) or not (w/o) chloroquine (CQ; C6628; Sigma, St. Louis, MO, USA, 20 μM) 24 h before flow cytometry. Then, 500 nM LTDR and Cyto-ID staining solution (ENZ-51031-0050; Enzo Life Sciences) were used for 30 min. [MFI Cyto-ID (w/CQ)/MFI LTDR (w/CQ)] − [MFI Cyto-ID (w/o CQ)/MFI LTDR (w/o CQ)] is autophagic flux [87].

### 4.8. Immunofluorescence

Senescent fibroblasts were treated with DMSO (0.01%) or ε-viniferin (4 µM) every 4 days for 12 days. Cells were then permeabilized with 0.1% Triton X-100 (X100RS; Sigma) in PBS for 15 min at room temperature (RT) and fixed with 4% paraformaldehyde (252549; Sigma) in PBS for 15 min. For 1 h at RT, blocking was carried out using PBS containing 10% FBS. Following the addition of primary antibodies, the samples were incubated overnight at 4 °C. Cells were incubated with secondary antibodies for 1 h at RT following three rounds of washing with ice-cold PBS. The ProLong Gold Antifade reagent (P36934; Invitrogen) was used to mount the samples after the nuclei were stained with DAPI (R37606; Invitrogen). A Carl Zeiss LSM 700 confocal microscope (Carl Zeiss, Oberkochen, Germany) was used to take pictures. Table 3 lists the primary and secondary antibodies used for immunofluorescence.

### 4.9. Senescent Associated β–Galactosidase (SA-β-gal) Staining

Senescent fibroblasts were treated with DMSO (0.01%) or ε-viniferin (4 µM) every 4 days for 12 days. As directed by the manufacturer, SA-β-gal staining was carried out (9860; Cell Signaling Technology, Beverly, MA, USA). Briefly, cells were fixed in 3% formaldehyde for 5 min and then stained overnight at 37 °C with freshly prepared SA-β-gal staining solution. The percentage of SA-β-gal-positive cells among total cells in randomly selected fields was calculated.

### 4.10. Quantitative PCR (qPCR)

Senescent fibroblasts were treated with DMSO (0.01%) or ε-viniferin (4 µM) every 4 days for 12 days. The RNase Mini Kit (74104; QIAGEN, Hilden, Germany) was used to extract total RNA from 1 × 10^6^ cells in accordance with the manufacturer’s instructions. After that, the DiaStarTM RT Kit (DR22–R10k; Solgent, Daejon, Republic of Korea) was used to reverse-transcribe the total RNA. Using a CFX ConnectTM Real-Time PCR Detection System (Bio-Rad, Hercules, CA, USA), qPCR was carried out using Solg^TM^ 2× Real-Time PCR Smart Mix (SRH83-M40h; Solgent). qPCR was performed using the following primer: 5′-CGCAATGGTGAAGGTC-3′ (*GAPDH*-forward) 5′-CGCCAGCATCACCCC-3′ (*GAPDH*-reverse), 5′-TTCACAAATCAGAGCTGGGCTGC-3′ (*RGS16*-forward), and 5′-CAGGTTCTCCTCACTGAACTCTG-3′ (*RGS16*-reverse). After denaturation at 94 °C for 5 min, qPCR was performed by repeating 40 cycles of 94 °C for 30 s, 57 °C for 30 s, and 70 °C for 10 s. The relative expression of *RGS16* RNA was normalized using the mean value of *36B4* RNA. Then, the relative expression of *RGS16* RNA was calculated using the 2^−ΔΔCt^ method. All samples were analyzed in biological triplicate.

### 4.11. Transcriptome Expression Profiling

Senescent fibroblasts were administered DMSO (0.01%) or ε-viniferin (4 µM) at 4-day intervals for 12 days. For transcriptome expression profiling, total RNA was extracted using the RNase Mini Kit (74104; QIAGEN, Hilden, Germany). Illumina platform using TruSeq Small RNA was used to find the expression (paired-end) of the generated transcripts. Contamination artifacts were removed to improve the analysis of the raw reads obtained during the sequencing process. HISAT2 (version 2.1.0; Johns Hopkins University Center for Computational Biology, Baltimore, MD, USA) was used to map the cleaned reads to the Homo sapiens (GRCh38, NCBI_109.20200522) genome. Then, the transcriptome was assembled using StringTie (version 2.1.3b; Johns Hopkins University Center for Computational Biology). Gene set enrichment analysis was performed to identify the differentially expressed genes. All samples were analyzed in biological triplicate. A *p*-value less than 0.05 was used as the statistical criterion to determine statistical significance.

### 4.12. Lenti–Viral Production and Infection

Using Lipofectamine 2000 (11668019; Invitrogen), 8 μg of plasmids (*pLenti-control* and *pLen-ti-RGS16*), 4 μg of VSV.G, and 4 μg of PAX2 were transfected into HEK 293T cells. After 24 h, the viral supernatant was harvested. The viral supernatant was mixed with Polybrene (TR–1003–G; 8 μg/mL; Millipore, Burlington, MA, USA). As previously described, senescent fibroblasts were infected [88].

### 4.13. Statistics

In the experiments measuring OCR and ECAR, data were collected through five biological replicates. Except for the OCR and ECAR experiments, data were collected through three biological replicates. Statistical analysis was performed using GraphPad Prism 10 (San Diego, CA, USA). Shapiro–Wilk and Levene tests were performed to test for normality and equality of variances, respectively. When the assumptions of normality and equality of variances were not met, the nonparametric alternative, the Mann–Whitney U test, was used. When the assumptions of normality and equality of variances were met, a two-way ANOVA followed by Bonferroni’s post hoc test was used. The type of error was shown as a standard deviation. A *p*-value less than 0.05 was used as the statistical criterion to determine statistical significance.

## Figures and Tables

**Table 1:** Secondary metabolites used for screening.

Compound Name	Structure	Bioactivity
Phillyrin	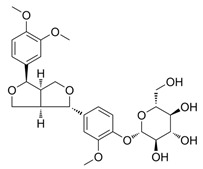	anti-inflammatory and anti-tumor activities [16]
Rosamultin	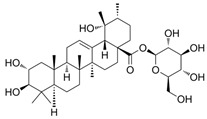	antioxidant properties [17]
ε-viniferin	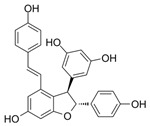	antioxidant properties [18]

**Table 2:** Information on the analysis of MMP, mitochondrial mass, and lysosomal mass.

Analysis	Dye Name	Company Name	Catalogue Number	Concentration
MMP	JC-10	Enzo Life Sciences, Farmingdale, NY, USA	ENZ-52305	0.6 µg/mL
Mitochondrial mass	MitoTracker^TM^ Deep Red FM Dye	Invitrogen, Waltham, MA, USA	M46753	50 nM
Lysosomal mass	LysoTracker^TM^ Deep Red (LTDR)	Invitrogen	L7526	500 nM

**Table 3:** Information on primary and secondary antibodies used in the immunofluorescence.

Analysis	Antibody Name		Company Name	Catalogue Number	Dilution in PBS
OXPHOS staining	primary	mouse anti-OXPHOS cocktail antibody	Abcam, Cambridge, Cambridgeshire, UK	ab110411	1:200
	secondary	Alexa Fluor^®^ 647 goat anti-mouse IgG antibody	Invitrogen	A-28181	1:200
LC3B staining	primary	rabbit anti-LC3B antibody	Abclonal, Boston, MA, USA	A19665	1:200
	secondary	Alexa Fluor^®^ 488 goat anti-rabbit IgG antibody	Invitrogen	A-11008	1:200

## Data Availability

The original contributions presented in this study are included in this article and supplementary materials; further inquiries can be directed to the corresponding authors.

## Supplementary Materials

The following supporting information can be downloaded at https://www.mdpi.com/article/10.3390/ph18091254/s1, Table S1. List of 31 genes significantly changed by more than 2 folds (ε-viniferin group versus DMSO group). Figure S1. Full-size image of immunofluorescence in Figure 3A and B. (A) Original image of DMSO (without chloroquine) in Figure 3A. (B) Original image of ε-viniferin (without chloroquine) in Figure 3A. (C) Original image of DMSO (witho chloroquine) in Figure 3B. (D) Original image of ε-viniferin (with chloroquine) in Figure 3B. Figure S2. ^1^H–NMR spectrum (600 MHz, Pyr-d_5_) of phillyrin isolated from *Osmanthus fragrans* var. aurantiacus. Figure S3. ^13^C–NMR spectrum (150 MHz, Pyr-d_5_) of phillyrin isolated from *Osmanthus fragrans* var. aurantiacus. Figure S4. ^1^H–NMR spectrum (600 MHz, CD_3_OD) of rosamultin isolated from *Rosa rugosa.* Figure S5. ^13^C–NMR spectrum (150 MHz, CD_3_OD) of rosamultin isolated from *Rosa rugosa*. Figure S6. ^1^H–NMR spectrum (600 MHz, CD_3_OD) of ε-viniferin isolated from *Vitis amurensis*. Figure S7. ^13^C–NMR spectrum (150 MHz, CD_3_OD) of ε-viniferin isolated from *Vitis amurensis*.
