# Supplementary material for: ε-Viniferin Rejuvenates Senescence via RGS16 Regulation: In Vitro Evidence
Source: Pharmaceuticals (Basel). 2025 Aug 24;18(9):1254. doi: 10.3390/ph18091254 (PMC12472759; doi:10.3390/ph18091254)
Supplement: Supplementary file 1 [file pharmaceuticals-18-01254-s001.zip › pharmaceuticals-3783743-supplementary.pdf]

Supplementary information

Table S1. List of 31 genes significantly changed by more than 2 folds (ε-viniferin group versus DMSO group).

Accession Number	Gene symbol	Description	Relative expression (vitisin B/DMSO)
NM_001204478,NR_037924	TVP23C-CDRT4	TVP23C-CDRT4 readthrough	-18.824933
NR_073179,NR_073180	LIPE-AS1	LIPE antisense RNA 1	-3.689750
gene-RN7SL608P	RN7SL608P	RNA, 7SL, cytoplasmic 608, pseudogene	-3.266855
NM_003761,XM_017005170	VAMP8	vesicle associated membrane protein 8	-3.035902
NR_135192	LOC101928595	uncharacterized LOC101928595	-2.642928
NM_001204848,NR_144937	TNFAIP8L2-SCNM1	TNFAIP8L2-SCNM1 readthrough	-2.487325
NR_146292,NR_146293	TDRKH-AS1	TDRKH antisense RNA 1	-2.402754
NM_024734,XM_011537158, XM_011537159,XM_017021646,XM_017021647,XR_001750558,XR_245721,XR_429330, XR_429332	CLMN	calmin	-2.394099
NM_004125	DNAJC25-GNG10	DNAJC25-GNG10 readthrough	-2.325815
XR_001748097	LOC105379880	uncharacterized LOC105379880	-2.267333
NM_153612,XM_006715379, XM_011535588,XM_017010470,XM_017010471,XM_017010472,XM_017010473,XM_017010474	HS3ST5	heparan sulfate-glucosamine 3-sulfotransferase 5	-2.227943
gene-ADGRF5P1	ADGRF5P1	adhesion G protein-coupled receptor F5 pseudogene 1	-2.216263
NM_001276713,XM_011543618,XM_011543623,XM_017009814,XM_017009815,XM_017009816,XM_017009817	ANKDD1B	ankyrin repeat and death domain containing 1B	-2.203859
XR_001746041,XR_001746042,XR_001746043,XR_001746044,XR_001746045,XR_0017	LOC105375721	uncharacterized LOC105375721, transcript variant X4	-2.158399

46046,XR_001746047,XR_001746048,XR_001746049,XR_001746050			
NM_001198910	CCDC169-SOHLH2	CCDC169-SOHLH2 readthrough	-2.150288
NM_001025081,NM_001025090,NM_001025092,NM_001025100,NM_001025101,NM_002385,XM_017025778,XM_017025780,XM_024451185,XM_024451186,XM_024451187,XM_024451188,XM_024451189,XR_001753201,XR_001753202	MBP	myelin basic protein	-2.143923
XR_001738082,XR_001738083,XR_001738084,XR_001738085,XR_001738086,XR_001738087,XR_001738088,XR_001738089,XR_001738090,XR_947417	LOC105378756	uncharacterized LOC105378756, transcript variant X2	-2.094314
NR_120398	LOC101060553	uncharacterized LOC101060553	-2.086372
NM_001114171,NM_006732, XM_005258691	FOSB	FosB proto-oncogene, AP-1 transcription factor subunit	2.003332
NM_001102654,NM_002527, XM_011520963	NTF3	neurotrophin 3	2.033552
NM_004962	GDF10	growth differentiation factor 10	2.162473
NM_014146,NM_032463,NM_032464,XM_011516558	LAT2	linker for activation of T cells family member 2	2.275955
gene-LOC390933	LOC390933	PAT1 homolog 1, processing body mRNA decay factor pseudogene	2.334615
NR_125817	LOC101929470	uncharacterized LOC101929470	2.419684
NM_002928,XM_024448796	RGS16	regulator of G protein signaling 16	2.560352
NM_001206942,NM_001206943,NM_001206944,NM_001206945,NM_006574	CSPG5	chondroitin sulfate proteoglycan 5	2.644677
NM_000236,XM_005254372, XM_005254374,XM_006720502,XM_017022176,XM_024449916,XM_024449917	LIPC	lipase C, hepatic type	2.781152

NR_034099	LINC01118	long intergenic non-protein coding RNA 1118	2.829079
gene-ARL4AP5	ARL4AP5	.	3.419872
NM_001289933	ZASP	ZO-2 associated speckle protein	5.755266
NR_040582	STAG3L3	stromal antigen 3-like 3 (pseudogene)	6.841783

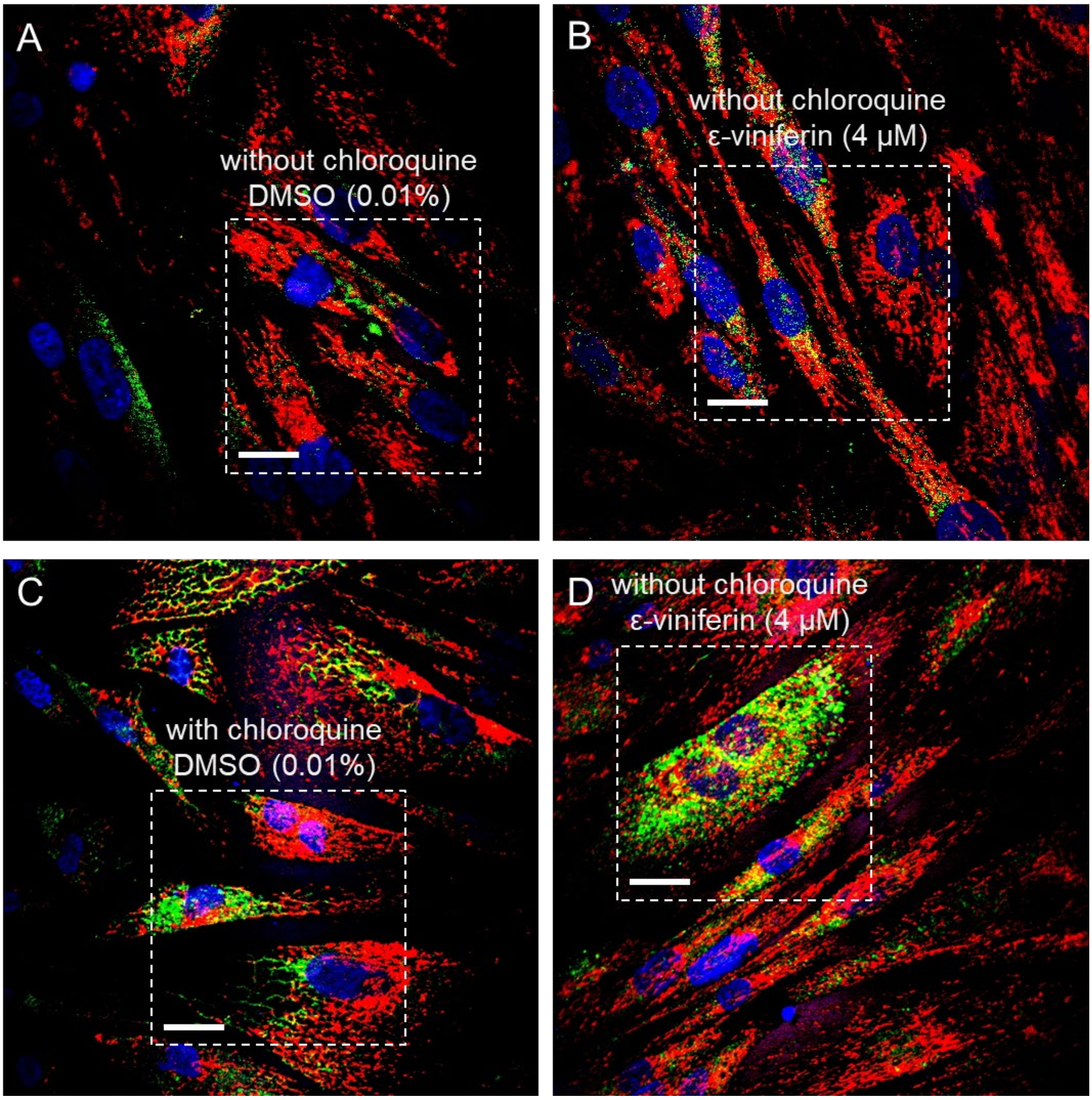

Figure S1. Full-size image of immunofluorescence in Figure 3A and B. (A) Original image of DMSO (without chloroquine) in Figure 3A. (B) Original image of ε-viniferin (without chloroquine) in Figure 3A. (C) Original image of DMSO (witho chloroquine) in Figure 3B. (D) Original image of ε-viniferin (with chloroquine) in Figure 3B.

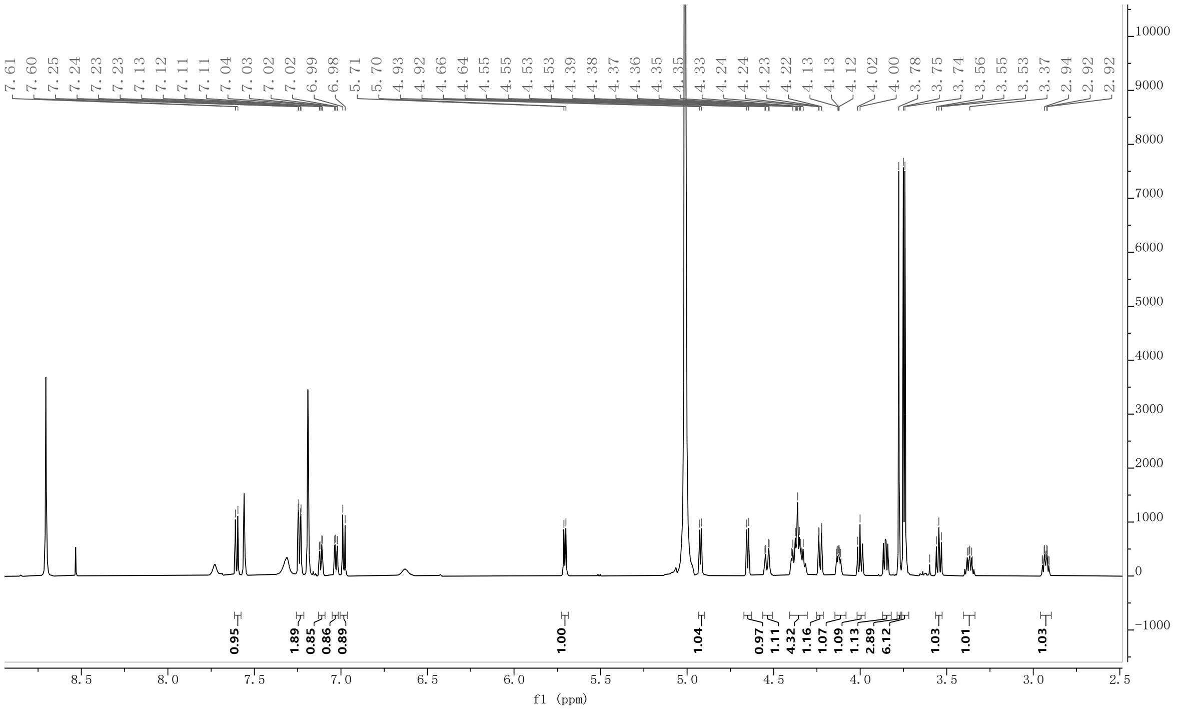

Figure S2. ^{1}H–NMR spectrum (600 MHz, Pyr-d_5) of phillyrin isolated from *Osmanthus fragrans* var. aura ntiacus.

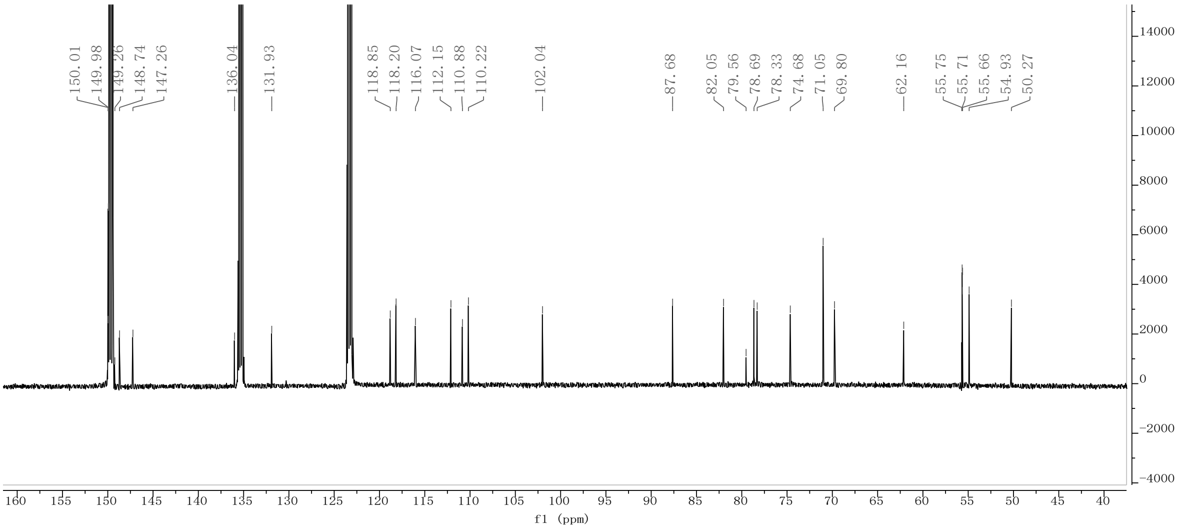

Figure S3. ^{13}C–NMR spectrum (150 MHz, Pyr-d_5) of phillyrin isolated from *Osmanthus fragrans* var. aura ntiacus.

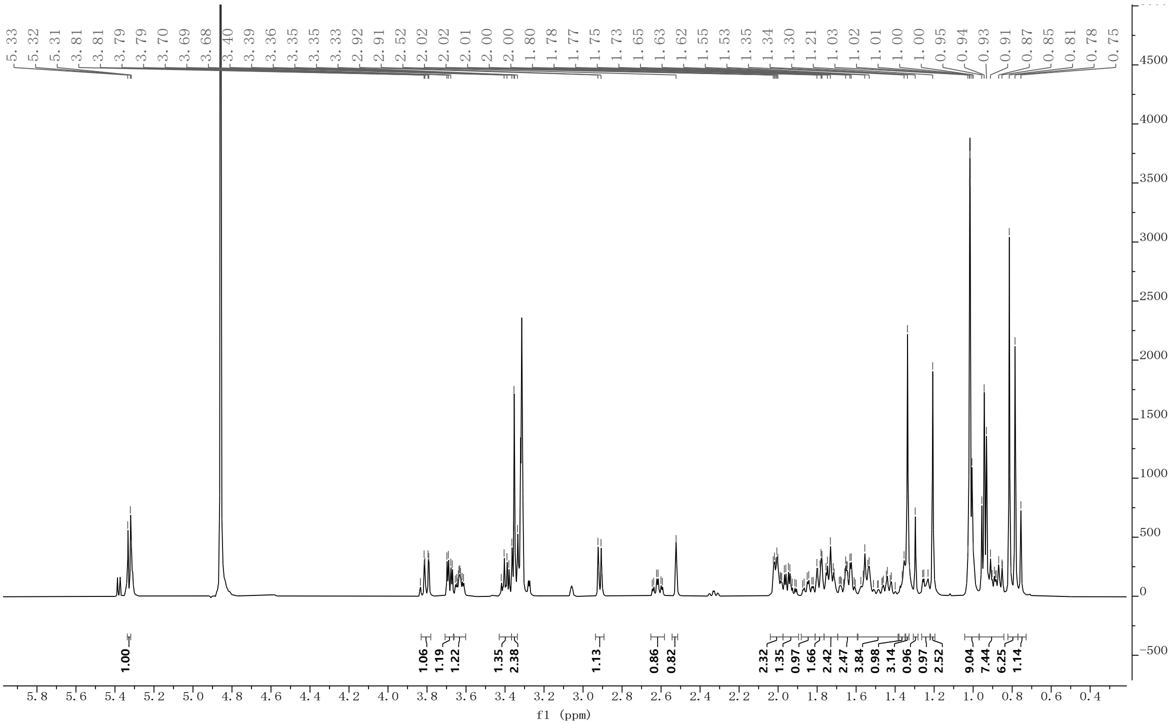

Figure S4. ^{1}H–NMR spectrum (600 MHz, CD_3OD) of rosamultin isolated from *Rosa rugosa*.

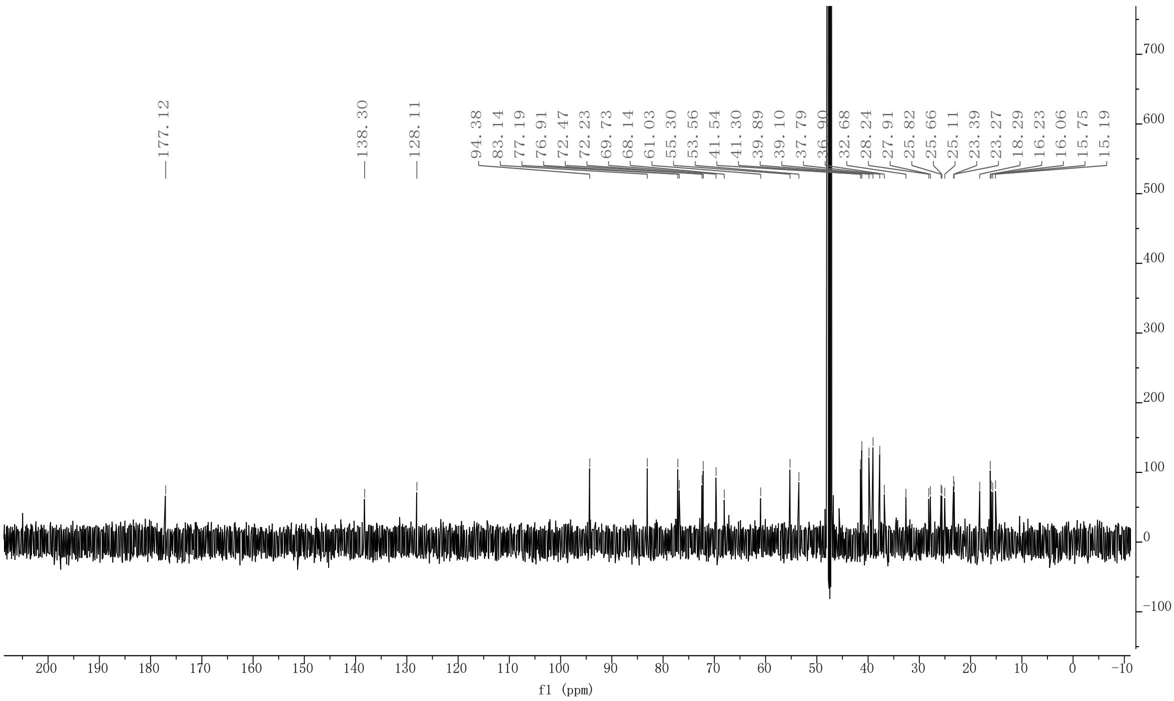

Figure S5. ^{13}C–NMR spectrum (150 MHz, CD_3OD) of rosamultin isolated from *Rosa rugosa*.

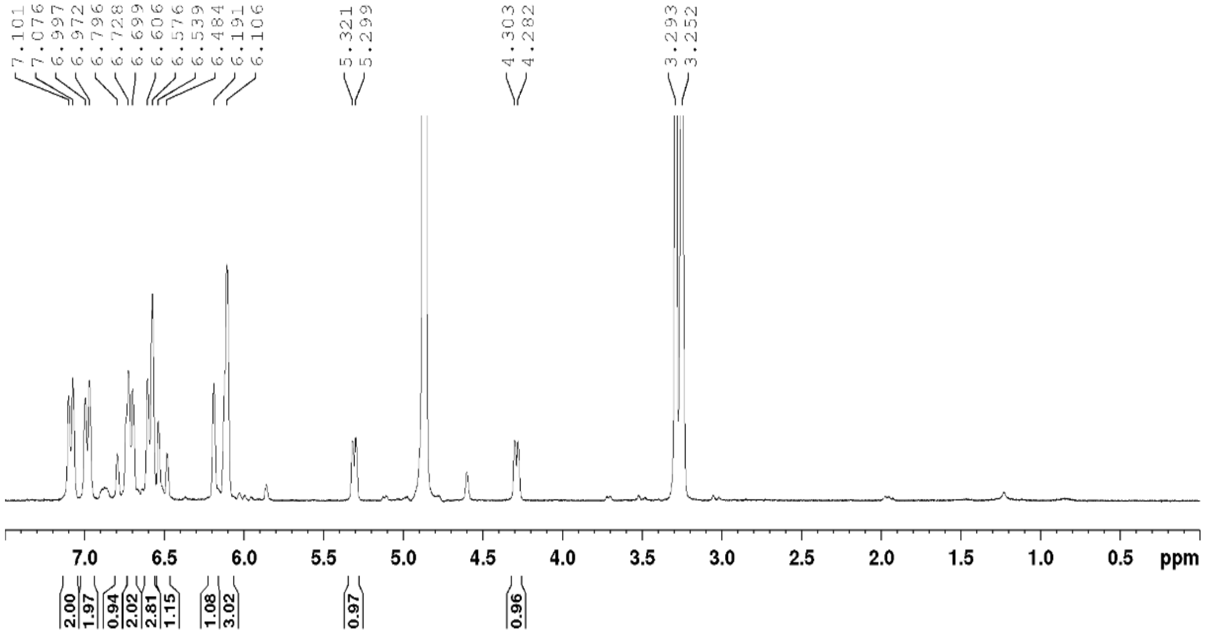

Figure S6. ^{1}H–NMR spectrum (600 MHz, CD_3OD) of ε-viniferin isolated from *Vitis amurensis.*

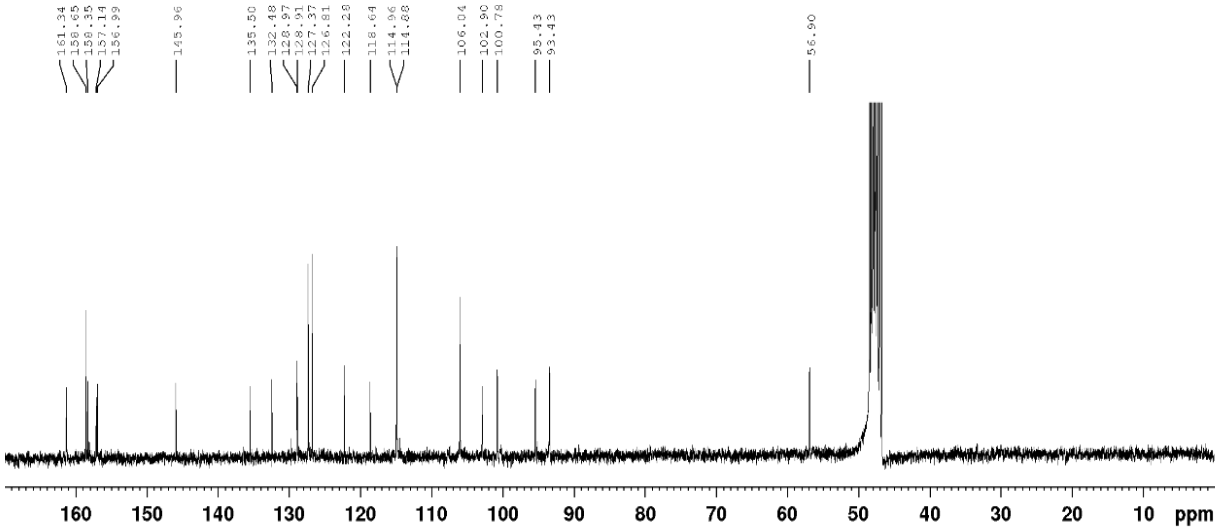

Figure S7. ^{13}C–NMR spectrum (150 MHz, CD_3OD) of ε-viniferin isolated from *Vitis amurensis*.
